# Supplementary material for: Effects of Channel Length Scaling on the Electrical Characteristics of Multilayer MoS2 Field Effect Transistor
Source: Micromachines (Basel). 2023 Jan 20;14(2):275. doi: 10.3390/mi14020275 (PMC9963916; doi:10.3390/mi14020275)
Supplement: Supplementary file 1 [file micromachines-14-00275-s001.zip › micromachines-2066193-supplementary.pdf]

micromachines

MDPI

Supplementary Materials

Effects of Channel Length Scaling on the Electrical Characteristics of Multilayer MoS_2 Field Effect Transistor

Sreevatsan Radhakrishnan [1], Suggula Naga Sai Vishnu [1], Syed Ishtiyaq Ahmed [1] and Rajagopalan Thiruvengadathan [1,2,*]

1 SIERS Research Laboratory, Department of Electronics and Communication Engineering, Amrita School of Engineering, Amrita Vishwa Vidyapeetham, INDIA, Coimbatore 641112, India
2 Mechanical Engineering Program, Department of Engineering and Technology, Southern Utah University, Cedar City, UT 84720, USA
* Correspondence: rajagopalanthiruveng@suu.edu

Description on NEGF Formulation

Consider a channel represented by it Hamiltonian(H), the contact terminals representing, source (S) and drain (D) with individual fermi levels μ_S and μ_D, respectively. The Hamiltonian H corresponds to the total energy of that system. Its spectrum consists of the set of energy eigenvalues, is the set of outcomes obtainable from a measurement of the system's total energy. Applied potential between the two contacts determines the difference of the Fermi levels μ_S and μ_D, thermal distribution of electron in through the Fermi function, $f_{S/D}$, Self-energy matrices, $\sum_S$ and $\sum_D$ describe coupling between the channel and the S/D contacts. This self-energy represents the contribution to the particle's energy or effective mass, due to interactions between the particle and its environment. The fermi levels correspond to occupancy factor of electron levels. Knowing H, μ_S, μ_D, $\sum_S$, and $\sum_D$, we can compute the retarded Green's function [1] as

$$G = [EI - H - \textstyle\sum_S - \sum_D]^{-1}$$

where EI is the matrix of energy of electron in channel and eigenvalues of H.

Hamiltonian :
The Hamiltonian H is given by [1]:

$H = H_0 + U$ where U indicates the potential caused by bias voltages and H_0 is band structures. In this section, the procedure to arrive at the Hamiltonian matrix [H_0] will be illustrated to describe the electronic bands of semiconductor channel in transistors.

- Effective Mass Hamiltonian

The complexity of band structure of MoS_2 makes it hard to describe H of the material. Effective mass can be adapted because its description around conduction band edge can be approximated to be parabola. This relation can describe the conduction band minimum as

$$\boldsymbol{E(k) = E_C + \frac{h^2k^2}{2m_c}}$$

where $\boldsymbol{m_c}$ ***and*** $\boldsymbol{E_C}$ are effective mass of the electron and conduction band, respectively. A differential equation which has same energy eigenvalues can be expressed like this [1]:

$$\boldsymbol{\left[E_C - \frac{h^2}{2m_c}\nabla^2\right] f(\vec{r}) = Ef(\vec{r})}$$

with any $f(\vec{r}) = exp(i\vec{k}.\vec{r})$ are eigenfunctions of this differential equation. The corresponding eigenvalues of the eigenfunctions are $E(\vec{k}) = E_C + \frac{h^2k^2}{2m_c}$. By FEM, this is converted into a Hamiltonian matrix H_0 that is given by:

$$H_0 = \begin{bmatrix} 2t_0 & -t_0 & \cdots & 0 & 0 \\ -t_0 & 2t_0 & & 0 & 0 \\ 0 & & & 2t_0 & -t_0 \\ 0 & 0 & \ddots & -t_0 & 2t_0 \\ 0 & 0 & & & \end{bmatrix}$$

where "a" is the lattice spacing and N is no. of lattices.

- Tight-binding model

The Tight-binding (TB) model is based on for E-K band structure, by superposition of wave functions of isolated atoms to arrive at approximate wavefunctions. If, a unit cell connected to 'm' unit cells around it, referred as neighbour unit cells. Matrix [H_{mn}]of size (b x b) describes connection between the two-unit cells, where b is the number of basis functions per unit cell.

The overall matrix equation is given by [1]:

$$\sum_m [\boldsymbol{H_{nm}}]\{\boldsymbol{\varphi_m}\} = \boldsymbol{E}\{\boldsymbol{\varphi_n}\}$$

where $\{\boldsymbol{\varphi_m}\}$ is a (b x 1) column vector that describes the wavefunction of the unit cell 'm'. If solution of this equation is $\{\boldsymbol{\varphi_m}\} = \{\boldsymbol{\varphi_0}\}\boldsymbol{e}^{\boldsymbol{i}\,\vec{\boldsymbol{k}}\,\cdot\, d_m}$ and based on the periodicity of the lattice, this equation becomes

$$\boldsymbol{E}(\boldsymbol{\varphi_0}) = \boldsymbol{E}(\vec{\boldsymbol{k}})\boldsymbol{\varphi_0} \text{ where } \boldsymbol{E}\,(\vec{\boldsymbol{k}}) = \sum\nolimits_{\boldsymbol{m}}[\boldsymbol{H_{mn}}]\,\boldsymbol{e}^{\boldsymbol{i}\vec{\boldsymbol{k}}\,(\overrightarrow{d_m}-\overrightarrow{d_n})}$$

The result of this equation is arrived by computing across all the coupling links to its neighbouring unit cells. The size of $[\boldsymbol{E}(\vec{\boldsymbol{k}})]$ is same as that of H_{nm}, b x b, and is used in plotting the band structure. By using any numerical package, the eigenvalues and eigen functions of $\boldsymbol{E}(\vec{\boldsymbol{k}})$is obtained and these eigenvalues become energy levels in bandstructure for each value of $\vec{\boldsymbol{k}}$. On contrary, a collection of such matrices [H_{nm}] make up the matrix [H_0] representing a periodic solid, which is of size (Nb x Nb), N being the total number of unit cells. This model enables us to calculate H_0 and offers accurate details of band structure. This calculated H_0 is employed to run self-consistent simulation in NEGF formalism as mentioned earlier.

Self-Energy:

The contact self-energy matrices calculated from contact surface Green's function, $g_s = ((E + \varphi_{0,L})I - H - \tau^* g_s \tau)$ which is solved iteratively. Her φ_0 and φ_L are the boundary potentials for the particular barrier height and drain bias and τ represents the contact coupling matrix.

Further, for ballistic transportation, the local density of states (LDOS) is represented using advanced Green's function as

$[A] = i[G - G^*] = [A_S] + [A_D]$,where $A_{S/D} = G\Gamma_{S/D}G^*$ and $\Gamma_{S/D} = i[\sum_{S/D} - \sum_{S/D}^*]$ describes level broadening due to contact. Herein, "*" represents the complex conjugate of the given Then the density matrix [41]is given by correlation function and is described as

$$[G^n] = [G\Gamma_S G^*]f_S + [G\Gamma_D G^*]f_D$$

Current at source or drain terminal per spin [1] is calculated as,

$$I = \frac{-2q}{h}\int_0^{+\infty} dE_k\,[f_0(E_k - \mu_1) - f_0(E_k - \mu_2)] = \frac{-2q}{h}[\mu_1 - \mu_2]$$

Alternatively, the current can be expressed as,

$$I = \frac{-e}{h}\int_{-\infty}^{+\infty} \hat{I}_{S/D}(E)dE$$

where $\hat{I}_{S/D} = Trace[\Gamma_{S/D}A]f_{S/D} - Trace[\Gamma_{S/D}G^n]$, h and e are Planck's constant and electron charge, respectively.

Poisson equation using FDM method:

Poisson equation is solved by using the finite difference method for a single back gate MOSFET structure. Using Gauss's law, the Poisson equation can be used to perform numerical analysis [2].

$$\oiint [\varepsilon \vec{E}(x, z\vec{E})] \,. d\vec{S} = \int_{\Omega} e[p - n + N_D - N_A] \, d\Omega$$

where ε is dielectric constant as function of coordinate, $\vec{E}$ is the electric field, p is the hole concentration, n is the electron concentration, N_D and N_A are donor and acceptor concentrations, respectively, e is charge of free electron. All internal nodal equations are arrived from Gauss's law, while the equations for nodes at interface are arrived by applying boundary conditions. The spatial derivatives for representing an internal node at [m, n], the finite difference method (FDM) is employed. By substituting $\vec{E} = -\nabla V$, the discretized linear finite difference equation of Gauss law is

$$\frac{a}{b}V_{m-1,n} + \frac{b}{a}V_{m,n-1} - 2\left(\frac{a}{b} + \frac{b}{a}\right)V_{m,n} + \frac{b}{a}V_{m,n+1} + \frac{a}{b}V_{m,n+1} = -\frac{ab}{\varepsilon}e(N_D - N_A - n)_{m,n}$$

where a and b are spacings along X and Z directions. $\varepsilon = \varepsilon_{ox}/\varepsilon_{ch}$ if node [m, n] is oxide/channel regions. If the node is located at the channel/oxide interface, the FDM equation becomes

$$\frac{a}{b}V_{m-1,n} + \frac{b}{2a}\left(1 + \frac{\varepsilon_{Bot}}{\varepsilon_{Top}}\right)V_{m,n-1} - \left(\frac{a}{b} + \frac{b}{a}\right)\left(1 + \frac{\varepsilon_{Bot}}{\varepsilon_{Top}}\right)V_{m,n} + \frac{b}{2a}\left(1 + \frac{\varepsilon_{Bot}}{\varepsilon_{Top}}\right)V_{m,n+1} + \frac{a}{b}\frac{\varepsilon_{Bot}}{\varepsilon_{Top}}V_{m+1,n} = -\frac{ab}{\varepsilon_{Top}}e(N_D - N_A - n)_{m,n}$$

where ε_{Bot} and ε_{Top} indicates dielectric constants for the materials above and below the interface.[2]

This expression shows that voltage of $V_{m,n}$ is solely determined by charge at [m, n] node and the voltage at the four nearest neighbours. This cluster is called computational molecule and is termed as the five-pointed star.[3]

Two of the common forms of boundary condition are:

- Dirichlet
- Neumann

The former is written as, $V(\mathbf{r}) = f(\mathbf{r})$ $(r \in \Omega_D)$ where Ω_D is the nodes satisfying the Dirichlet condition.[3] This behaves as a forced solution to the function f at given points. For gate terminal, substituting Ω_D with nodes at gate-oxide interfaces, the numerical equation can be easily written as, $V_{m,n} = V_G$ where V_G is the gate voltage.

On the other hand, the Neumann boundary condition can be used if derivative is known for a given function f, which is defined as:

$\frac{\partial V(r)}{\partial n} = f'(n)$ $r \in \Omega_N$ where N is the unit normal vector, and $f'(n)$ is the set of available derivatives.[3] If this, $f'(n)$ is fixed to 0.0 V/m,

$V_{m,n} - V_{m\pm1,n} = 0$ for the top and bottom edges

$V_{m,n} - V_{m,n\pm1} = 0$ for the left and right edges

$2V_{m,n} - (V_{m+1,n} + V_{m,n\pm1}) = 0$ for the two corner nodes along the top edge and

$2V_{m,n} - (V_{m-1,n} + V_{m,n\pm1}) = 0$ for the two corner nodes along the bottom edge.

The solution over all voltage nodes $V_{m,n}$ can be represented as a simple matrix equation as the node is solely dependent on its four nearest neighbours. Let the vector x containing all the voltage samples in the domain can be described as [3],

$$x = [V_{1,1}\ V_{1,2}\ V_{1,3}\ \ldots\ldots\ V_{m,n-1}\ \ V_{m,n}\ V_{m,n+1}\ \ldots\ldots\ \ V_{N_Z,N_X}]^T$$

we can write entire problem as a matrix equation form: $Ax = b$ where b is a vector contains information about the charge densities as well as the boundary conditions. Therefore, solution vector x is computed by matrix inversion and is given by: $x = A^{-1}b$. Newton-Raphson method is used to arrive at a convergent solution.

References

1. Datta, S. Quantum Transport: Atom to Transistor; Cambridge University Press, Cambridge, UK 2005; Volume 9780521631457, pp. 1–404.
2. Zhibin R. Nanoscale MOSFETs: Physics, simulation and design. Ph.D. Disseration, Purdue University, West Lafayette, IN, USA, 2001
3. Nagel, J.R. Finite-Difference Simulation of Eddy Currents in Nonmagnetic Sheets via Electric Vector Potential. IEEE Trans. Magn. 2019, 55, 1–8, https://doi.org/10.1109/tmag.2019.2940204.
